# Detection of airbag impact-induced cone photoreceptor damage by adaptive optics scanning laser ophthalmoscopy: a case report

**DOI:** 10.1186/s12886-016-0275-4

**Published:** 2016-07-08

**Authors:** Yoshihiro Kaizu, Shintaro Nakao, Muneo Yamaguchi, Yusuke Murakami, Hani Salehi-Had, Tatsuro Ishibashi

**Affiliations:** Department of Ophthalmology, Graduate School of Medical Sciences, Kyushu University, 3-1-1 Maidashi, Higashi-Ku, Fukuoka, 812-8582 Japan; Atlantis Eyecare, Huntington Beach, CA USA

**Keywords:** Motor vehicular accident, Photoreceptor damage, Scotoma, AO-SLO, Traumatic maculopathy

## Abstract

**Background:**

The purpose of this study was to report a case of traumatic maculopathy with para-central visual field defects following an impact by airbag deployment using adaptive optics scanning laser ophthalmoscopy (AO-SLO).

**Case presentation:**

A 51-year-old man was involved in a motor vehicular accident and his left eye was struck by the deployed airbag, resulting in a para-central scotoma. The patient underwent a full ophthalmologic examination, spectral-domain optical coherence tomography (SD-OCT), and imaging with prototype AO-SLO systems (Canon Inc.) at 14 and 22 months after the injury. Images focused on the photoreceptor layer were recorded in the foveal area, and a montage of AO-SLO images was created. On AO-SLO, focal dark areas could be observed in the left eye at 14 months after the injury. The analysis showed that the cone mosaic (cone density, 16503/mm^2^; ratio of hexagonal Voronoi domain, 36.3 %; average nearest-neighbor distance (NND)/expected NND, 0.606) was disordered compared with the normal area of the same eye (cone density, 24821/mm^2^; ratio of hexagonal Voronoi domain, 44.1 %; average NND/expected NND, 0.739). The cone defect area corresponded to the area of the scotoma. A second AO-SLO was performed on the patient at 22 months after the injury and although there were still areas with reduced cone reflectivity, partial improvement of cone mosaic was detected by AO-SLO at this time point.

**Conclusion:**

Partial recovery of damaged cone photoreceptors following closed globe blunt ocular trauma can be documented using AO-SLO longitudinal tracking.

## Background

Airbags have been developed to reduce injury and death from motor vehicle collisions. However, airbags have also been reported to cause various ocular injuries including commotio retinae [1] and maculopathy [2, 3]. Recently, Flatter et al. reported outer retinal structural abnormalities in nine subjects with visual field defects after closed globe blunt ocular trauma using adaptive optics scanning laser ophthalmoscopy (AO-SLO) [4]. However, there has been no study that has followed up a patient with an airbag-induced eye injury using AO-SLO. This article reports a follow-up of a case of traumatic maculopathy with para-central visual field defects following deployment of an airbag using AO-SLO longitudinal tracking.

## Case presentation

**Fig. 1:**
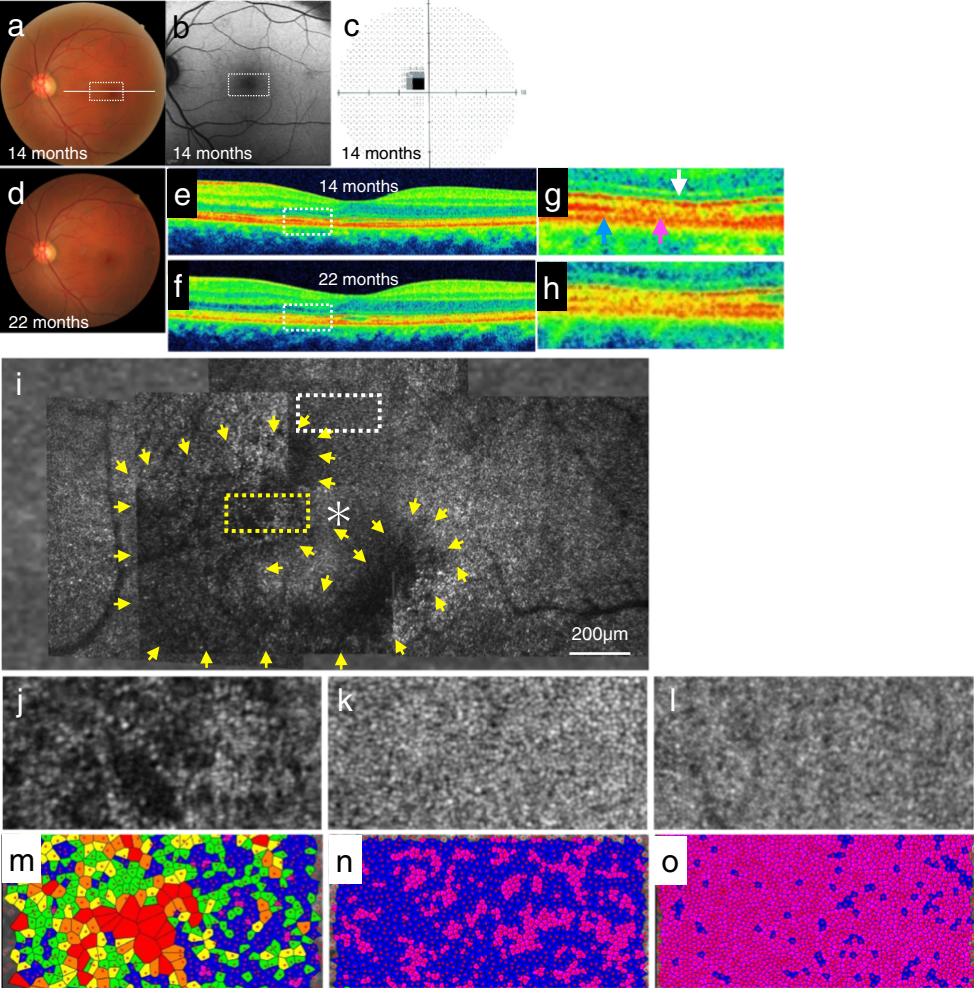
**a, b** The fundus examination and the fundus autofluorescence of the left eye was normal 1 year after the first clinical visit (14 months after the injury). White square and white line indicate AO-SLO image (**i**) and SD-OCT (**e**), respectively. **c** Humphrey visual field 10–2 showed a para-central blind spot in the left eye at 14 months after the injury. **d** The fundus photo was also normal at 22 months after the injury. **e, f, g, h** SD-OCT (**e, g**; 14 months, F, H; 22 months) showed a continuous but slightly depressed ellipsoid zone (*white arrow* in **g**). **g** and **h** show magnified images of the white dotted square in **e** and **f**, respectively. The blue arrow indicates the area of intact interdigitation zone, whereas the pink arrow indicates the area of disrupted interdigitation zone. **i** The AO-SLO montage (1 mm X 2 mm) of the fovea in the left eye showed an area with reduced cone reflectivity corresponding to the area of the scotoma (*yellow arrows*). Yellow and white dotted squares indicate AO-SLO image at the 0.25 mm nasal and 0.34 mm superior areas from the fovea, respectively, 14 months after the first visit. The asterisk indicates the central fovea. **j, k** AO-SLO images at the yellow dotted square (**j**) showed patchy reduced cone reflectivity. AO-SLO at the white dotted square (**k**) showed normal cone spacing. **m, n** Color maps of the cone mosaic in the white and yellow dotted squares (Red > 250 μm^2^, Orange = 200-250 μm^2^, Yellow = 150-200 μm^2^, Green = 150-200 μm^2^, blue = 100-150 μm^2^). The analysis revealed that the cone mosaic of the yellow dotted square (cone density, 16503/mm^2^; ratio of hexagonal Voronoi domain, 36.3 %; average NND/expected NND, 0.606) was disordered compared with the white dotted square (cone density, 24821/mm^2^; ratio of hexagonal Voronoi domain, 44.1 %; average NND/expected NND, 0.739). **l** AO-SLO image at the 0.25 mm nasal area in the 29-year healthy man. **o** Color map of cone mosaic of the imaged area in the 29-year-old man (cone density, 38750/mm^2^; ratio of hexagonal Voronoi domain, 43.1 %; average NND/expected NND, 0.718)

**Fig. 2:**
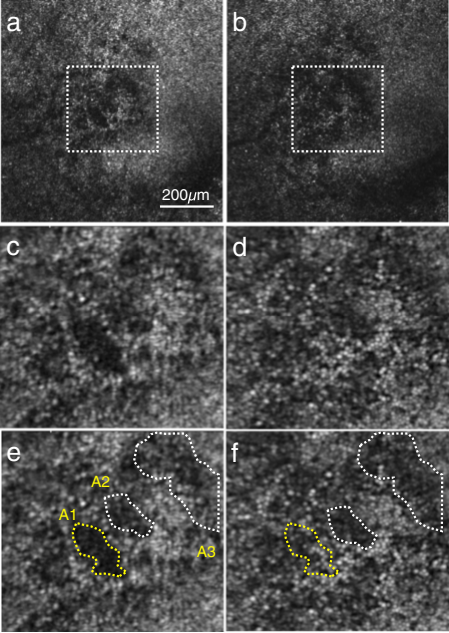
**a, b** AO-SLO images at the 0.25 mm nasal area from the fovea in the left eye showed an area with reduced cone reflectivity corresponding to the area of the scotoma at 14 (**a**) and 22 months (**b**) after being injured by the airbag. **c, d** The cone mosaic in the white dotted squares at 14 (**c**) and 22 months (**d**) after the injury. **e** 14 months after the injury. Cone densities at *A1* (*yellow dot circle*), *A2 (white ​dot circle)* and *A3 (white ​dot circle)* are 6647/mm^2^, 13202/mm^2^ and 16409/mm^2^, respectively. **f** 22 months after the injury, Cone densities at *A1 (yellow dot circle)*, *A2 (white ​dot circle)* and *A3 (white ​dot circle)* are 14359/mm^2^, 14490/mm^2^ and 15076/mm^2^, respectively. Cone mosaic at *A1 (yellow)* area was improved at 22 months as compared with 14 months after the injury, whereas cone mosaic did not show any apparent improvement or progression in *A2 and A3 (white)* areas during our observation

A 51-year-old man was involved in a motor vehicular accident and was struck in his left eye by the deployed airbag. He was wearing the seat belt. Following the impact from the deployed airbag, he immediately noticed a para-central fixed scotoma in his left eye. The patient had no neurological deficits and had no other notable injuries. He visited a community ophthalmologist. The anterior segment ophthalmoscopic findings were unremarkable bilaterally. The posterior segment examination revealed commotio retinae and several retinal hemorrhages around the optic nerve but not in the macula in his left eye. Two months after the injury, he was referred from the community ophthalmologist to Kyushu University department of ophthalmology due to the lack of improvement of his scotoma. At the initial visit, his visual acuity was 20/20 in each eye and his slit-lamp and fundus examinations were normal. Fundus photography and auto fluorescence did not show any apparent abnormalities (Fig. 1a and b). Commotio retinae and retinal hemorrhage were resolved at our first examination. Humphrey visual field 10–2 revealed a para-central scotoma in the left eye (Fig. 1c). SD-OCT (Cirrus HD; Carl Zeiss Meditec, Dublin, CA, USA) showed a continuous but slightly depressed ellipsoid zone and a discontinuous interdigitation zone band (Fig. 1e, g). During the follow-up period of 22 months, there was no improvement or worsening of his symptomatic scotoma. The visual acuity, repeated fundus examinations, SD-OCT and perimetry showed no significant changes or improvement over 22 months (Fig. 1d, f, h). A prototype AO-SLO system (Canon Inc., Tokyo, Japan) was used to examine his eyes at 14 and 22 months after the injury. On AO-SLO, significant cone photoreceptor defects could be observed in the left eye at 14 months after the injury (Fig. 1i). The area of cone defect corresponded to the area of the scotoma (Fig. 1c, i). Analysis revealed that the cone mosaic was disordered when compared with a normal area for this patient, and when compared with the corresponding area in a healthy male (cone density: 16503/mm^2^ vs 24821/mm^2^ vs 38750/mm^2^; ratio of hexagonal Voronoi domain: 36.3 % vs 44.1 % vs 43.1 %; and average nearest-neighbor distance (NND)/expected NND: 0.606 vs 0.739 vs 0.718, respectively; Fig. 1j-o). The cone density of the injured area was also apparently lower than that of the average cone density obtained from an extensive normative database. Furthermore, when AO-SLO images obtained at 22 months (Fig. 2b) were compared with AO-SLO images of the same area obtained at 14 months (Fig. 2a), areas with reduced cone reflectivity were still observed in the images obtained at 22 months. To compare the injured area at the cellular level between these two time points, we analyzed the cone density in several injured areas. The analysis showed that the cone density had increased at 22 months after the injury compared with 14 months after the injury in one of the areas examined (yellow A1, 14 months: 6647/mm^2^ vs 22 months: 14359/mm^2^). The cone density in the other areas examined (white; A2 and A3) was not significantly changed (white areas A2, 14 months: 13202/mm^2^ vs 22 months: 14490/mm^2^, A3, 14 months: 16409/mm^2^ vs 22 months: 15076/mm^2^) (Fig. 2c-f).

No clinical images were acquired immediately post trauma when the 51-year-old male patient presented to a non-institutional healthcare provider. The patient subsequently underwent full ophthalmologic examinations at Kyushu University Hospital, which included best-corrected visual acuity, slit-lamp examination, fundus examination, and color fundus photography. The pupil was first dilated with one drop of 0.5 % toropicamide and one drop of 0.5 % phenylephrine hydrochloride before fundus autofluorescence was acquired with a confocal scanning laser ophthalmoscope (HRA; Heidelberg Engineering GmbH, Dossenheim, Germany). High-density five line raster scans were obtained with an SD-OCT. The central fovea was defined as the location without the inner retinal layers in the macula region. A prototype confocal AO-SLO system developed by Canon was used. As a control, AO-SLO images were taken in a similar fashion at the 0.25 mm nasal area in a 29-year-old healthy man. Axial length was measured in each subject using IOL master (Carl Zeiss Meditec, Jena, Germany) to calculate the AO-SLO image scale. As previously described [5, 6], the prototype AO-SLO device has an AO system that is able to measure and correct aberrations of the subject's eyes, a high-resolution confocal SLO imaging system, and a wide-field imaging subsystem. The wavelength of AO-SLO was 845 nm, and the wavelength of the beacon light for the measurement of wave front aberrations was 760 nm. The imaging light and the beacon light power were set at 400 and 100 μW, respectively, in accordance with the safety limits set by the American National Standards Institute. Images were recorded for one second per scan area with a field size of 1.2 × 1.2° and 2.8 × 2.8°. The frame rate was 32 frames per second. The AO-SLO device was focused on the photoreceptor layer and these images showed individual cone photoreceptor cells. At 14 and 22 months after the injury, images focused on the photoreceptor layer were recorded in the foveal area corresponding to the scotoma and the surrounding area within the macula, and a montage of AO-SLO images was created semi-automatically using cones and blood vessels registration. The montage was done with almost cone-for-cone precision. The central fovea was defined as the location of the fixation point in the macula region. The central fovea from the foveal pit was also confirmed on OCT. The area of photoreceptor disruption 0.25 mm nasal to the fovea and the undisrupted region 0.34 mm superior to the fovea were chosen for the examination of photoreceptor metrics. All measurements were within 0.5 mm eccentricity. The same areas (the area of the scotoma and the surrounding area within the macula) were imaged with the AO-SLO at 14 and 22 months after the injury. Blood vessel registration was used to confirm that the images obtained at 14 and 22 months corresponded to the same area. For each cone mosaic, the cone density, ratio of hexagonal Voronoi domain, average NND/expected NND was examined to estimate the spatial organization of the cone mosaics as previously reported [7]. Voronoi domains were constructed for each cell by defining points in the regions that were closer to that cell than to any other cell in the mosaic. The ratio of hexagonal Voronoi domains is a representation of the regularity of cellular arrangement. The NNDs were determined by calculating the minimum distances from the center of that cell to the centers of every other cell in the mosaic. Expected NND was calculated as the expected value for a perfectly hexagonally packed mosaic with a density equal to that in each location.

## Discussion

The use of airbags in motor vehicles has become almost universal. It has saved many lives and reduced the incidents of facial trauma. However, it has been reported that airbag deployment can cause a variety of eye injuries [3]. The percentage of vitreoretinal injury in eyes injured by deployed airbags is approximately twenty percent [8]. A few papers have reported persistent scotomas associated with impact from a deployed airbag [4]. Manche et al. showed that the scotoma was attributed to a break in the Bruch’s membrane [9]. Carrim et al. indicated the presence of focal thinning of neurosensory retina on the OCT [10]. A recent report also showed retinal dysfunction of the injured eye with multifocal electroretinography following foveal detachment with an impending macular hole [2]. They suggested that the photoreceptor injury was caused by the sudden shock on impact. In this report, cone damage could be seen on AO-SLO and our observation supports the previous observations made by Carrim et al. Similar observations using a different AO-SLO system have also been reported by Flatter et al. [4]. These findings using two different AO-SLO systems confirmed that injury caused by a deployed airbag could cause photoreceptor damage. Therefore, it may be necessary to examine photoreceptors at the cellular level after impact with an airbag even in the absence of abnormalities based on fundus examination or conventional imaging modalities. In our case, SD-OCT showed a slightly curved ellipsoid zone and a discontinuous interdigitation zone. It could be argued that our AO-SLO system is unable to focus on the injured cone photoreceptor because of the depressed ellipsoid zone. However, the focus depth of our machine is around 60 μm, which is sufficient to focus on the entire length of cone photoreceptors [5]. This indicates that the dark area corresponds to damaged cone photoreceptors and is not an artifact.

We have recently reported areas with abnormal cone mosaic on AO-SLO that corresponded to areas of disrupted ellipsoid zone on SD-OCT in a patient with acute zonal occult outer retinopathy [7]. This observation suggests that images of cone mosaic on AO-SLO could be derived from the reflection of mitochondria in the ellipsoid zone. However, in this patient with a deployed airbag-induced injury, SD-OCT showed an intact ellipsoid zone with discontinuous interdigitation zone at the first AO-SLO examination. Therefore, both the ellipsoid zone and interdigitation zone could be important for obtaining normal cone mosaic images by AO-SLO.

In a recent paper by Flatter et al. using AO-SLO, similar cone damage with various closed globe blunt ocular trauma including three cases with deployed airbag-induced injury was reported [4]. Furthermore, Flatter et al. also presented two subjects with closed globe blunt ocular trauma that were followed up by AO-SLO [11]. In the present study, the course of the traumatic maculopathy was followed with our prototype AO-SLO at two time points after the injury. Interestingly, AO-SLO detected partial improvement of the cone mosaic in spite of the persistent scotoma. Several previous studies have shown temporal fluctuation in the reflectivity of individual cone photoreceptors [12]. In our case however, the change of reflectivity of the cone mosaic was island shaped, suggesting that the alteration is due to improved cone structure or function in spite of the persistent scotoma.

It is important to note that in this present case, the injured eye immediately post trauma could not be examined with AO-SLO. Therefore, further observations with AO-SLO will be necessary to evaluate the evolution of photoreceptor damage in the immediate aftermath of an injury caused by a deployed airbag. This study also has a few limitations concerning the controls used. AO-SLO images taken from a younger subject (29-year-old) was used as a control for the injured subject (51-year-old). Previous studies using AO-SLO have reported that cone-packing density decreases with increasing age within the foveal center [13], although in one study this difference was not statistically significant [14]. In addition, the AO-SLO image from the superior area was used as an internal control for the injured area. Although this was done within a very small retinal eccentricity, previous reports have shown small differences in cone packing density between nasal and superior areas [15, 16]. Finally, it is important to note the dearth of normative data on photoreceptor morphology using AO-SLO, despite our study and previous studies highlighting the ability of this imaging modality in distinguishing normal and abnormal photoreceptors [4].

In conclusion, we have been able to demonstrate that AO-SLO is a useful tool for the diagnosis and follow-up of eye injuries with traumatic maculopathy associated with deployed airbags. AO-SLO can indicate anatomically that cone photoreceptor injury is the cause of persistent central and para-central scotomas in deployed airbag-associated traumatic maculopathy and may be useful in assessing closed globe blunt traumas in general.

## Conclusion

This study reports a case of traumatic maculopathy with para-central visual field defects consequent to airbag deployment that was followed using AO-SLO. AO-SLO was able to indicate that the cone defect area corresponded to the area of the scotoma and could also detect partial improvement of the cone mosaic during the follow-up period.

## Abbreviations

AO-SLO, adaptive optics scanning laser ophthalmoscopy; NND, average nearest-neighbor distance; SD-OCT, spectral-domain optical coherence tomography
